# Long-term follow-up with a smartphone application improves exercise capacity post cardiac rehabilitation: A randomized controlled trial

**DOI:** 10.1177/2047487320905717

**Published:** 2020-02-28

**Authors:** Pernille Lunde, Asta Bye, Astrid Bergland, Jostein Grimsmo, Even Jarstad, Birgitta Blakstad Nilsson

**Affiliations:** 1 Department of Physiotherapy, Faculty of Health Sciences, OsloMet – Oslo Metropolitan University, Norway; 2 Department of Nursing and Health Promotion, Faculty of Health Sciences, OsloMet – Oslo Metropolitan University, Norway; 3 Regional Advisory Unit for Palliative Care, Department of Oncology, Oslo University Hospital, Norway; 4 Norwegian Heart and Lung Patient Organization, LHL-Hospital Gardermoen, Norway; 5 Norwegian Sport Medicine Clinic (NIMI), Department of Cardiology and Exercise Physiology, Norway; 6 Section for Physiotherapy, Division of Medicine, Oslo University Hospital, Norway

**Keywords:** MHealth, cardiac rehabilitation, mobile phone app, smartphone, lifestyle

## Abstract

**Background:**

Mobile health interventions, especially smartphone applications (apps), have been proposed as promising interventions for supporting adherence to healthy behaviour in patients post cardiac rehabilitation (CR). The overall aim of the study was to examine the effect of individualized follow-up with an app for one year on peak oxygen uptake (VO_2peak_) in patients completing CR.

**Design:**

The study was designed as a single-blinded multicentre randomized controlled trial.

**Methods:**

The intervention group (IG) received individualized follow-up enabled with an app for one year, while the control group (CG) received usual care. The primary outcome was difference in VO_2peak_. Secondary outcomes included exercise performance (time to exhaustion, peak incline (%) and peak velocity (km/h)), bodyweight, resting blood pressure, lipid profile, triglycerides, exercise habits, health-related quality of life, health status and self-perceived goal achievement.

**Results:**

In total, 113 patients completing CR (73.4% with coronary artery disease, 16.8% after valve surgery and 9.8% with other heart diseases) were randomly allocated to the IG or CG. Intention to treat analyses showed a statistically significant difference in VO_2peak_ between the groups at follow-up of 2.2 ml/kg/min, 95% confidence interval 0.9–3.5 (*p* < 0.001). Statistically significant differences were also observed in exercise performance, exercise habits and in self-perceived goal achievement.

**Conclusions:**

Individualized follow-up for one year with an app significantly improved VO_2peak_, exercise performance and exercise habits, as well as self-perceived goal achievement, compared with a CG in patients post-CR. There were no statistically significant differences between the groups at follow-up in the other outcome measures evaluated.

## Introduction

The beneficial effects of cardiac rehabilitation (CR) have been well demonstrated, and currently CR has a class IA recommendation in European guidelines on cardiovascular disease (CVD) prevention.^1,2^ Although continuation of healthy behaviours is necessary to improve long-term prognosis, adherence to healthy behaviours adapted during CR is challenging for many patients.^1^ The majority of heart patients do not achieve the guideline standard for secondary prevention, with a high prevalence of physical inactivity, persistent smoking and unhealthy diets; consequently, most patients are overweight or obese ≥6 months after a heart event.^3^ Research evaluating adherence to healthy behaviour after CR (post-CR) is, therefore, warranted.^1^

The main goal of CR and secondary prevention is to prevent subsequent heart events.^1,4^ Exercise capacity, measured as peak oxygen uptake (VO_2peak_), has been established in the last decades as an independent predictor of cardiovascular risk, cardiovascular death and all-cause mortality, both in healthy individuals^5–7^ and in patients with coronary artery disease (CAD).^8^ It is widely accepted that higher levels of VO_2peak_ are associated with better health outcomes as this predictor improves the overall CVD risk profile.^6^ Therefore, maintenance programmes post-CR should intend to maintain or improve VO_2peak_.

Digital health interventions have been proposed to meet challenges of adherence of healthy behaviour, and have, thus, been suggested as potential interventions post-CR.^1,9,10^ Mobile health (mHealth), defined as medical and public health practice supported by mobile devices,^11^ includes many of today’s digital health interventions, whereas the use of smartphone applications (apps) has been considered as particularly promising for secondary prevention due to their ability to monitor patients’ health from anywhere at any time.^12,13^ In a recent systematic review, the use of wearable physical activity monitors (including apps) has shown to improve exercise capacity to a greater extent compared with controls post-CR.^14^ Only three of the nine included studies in this systematic review evaluated VO_2peak_ with a follow-up time ranging from 12 weeks to six months.

To the best of our knowledge, no research exists on the effect of using an app for one year to promote and monitor adherence to healthy behaviours, post-CR. Therefore, the primary aim of this study was to examine whether individualized follow-up with an app for one year post-CR could improve VO_2peak_, compared with a control group (CG) that received usual care. Secondary, we aimed to evaluate the effect of individualized follow-up with an app for one year post-CR on exercise performance, bodyweight, resting blood pressure (BP), lipid profile, triglycerides, exercise habits, health-related quality of life (HRQL), health status and self-perceived goal achievement.

## Methods

### Setting and participants

Patients were recruited from two CR centres in the eastern part of Norway. Patients attending these centres are referred by a physician for rehabilitation after various forms of heart diseases. The most common referral causes are CAD and valve surgery. Patients may start CR two weeks after percutaneous coronary intervention and 6–8 weeks after open heart surgery. These CR centres offer, in total, three different CR programs: 12-week outpatient CR, four-week inpatient CR and one-week inpatient CR. Approximately one third of the patients were recruited from each of the three CR programs.

The inclusion criteria were as follows: patients completing CR at one of the three CR programs; age ≥40 years; owner and user of an Android or Apple smartphone; and able to read and understand Norwegian or English.

The exclusion criteria were as follows: ischemia or arrhythmias uncovered at cardiopulmonary exercise test (CPET) that gave restrictions equivalent to <80% of maximal heart rate or BORG scale (6–20) <15 at exercise. In addition, patients with muscular or skeletal disorders that affected exercise capacity more than the heart disease were excluded. Furthermore, patients with severe malignant disease – that is, advanced cancer – that affected the patient’s life span to a greater extent than their heart disease were excluded.

### Design

This study was a single-blinded, randomized controlled trial, comparing an intervention group (IG) with a CG. A computer-generated, permuted block randomization scheme was used to allocate the patients. The allocation ratio was 1:1 and randomization was stratified by the CR program. Patients were randomly allocated to one of the two groups via concealed allocation right after baseline assessment. The IG received individualized technology-based follow-up for one year, while the CG received usual care. The Regional Committee for Medical and Health Research Ethics (South-East ID: 2016-1476) approved the study protocol, and the study was conducted according to the Helsinki Declaration. All patients gave informed, written consent before inclusion in the study. The study protocol was registered in ClinicalTrials.gov (NCT03174106). Additionally, details of design, methods, sample size, randomization and organization have been previously published.^15^ Reporting follows the CONSORT 2010 statement.^16^

### Intervention

Patients in the IG received access to an app and teaching in how to use it right after baseline assessment. The app used was developed to guide and help individuals change behaviour and maintain habits. The app permitted the patient to set individual goals (supplementary Figure S1) with tasks and accompanying reminders. Goals and tasks decided during baseline assessment were added to the app, and each patient decided when and how often reminders of their tasks should appear. Additionally, patients could write notes related to each goal during the study period. The app itself provided automatic reminders and evaluations of tasks and weekly goal achievement. In these evaluations, the patients replied with a red or green face, depending on whether they had completed the planned task or not, and rated their weekly goal achievement on a scale from 0 to 100.

A supervisor had access to an administrator interface (supplementary Figure S2) and monitored the goals, tasks and notes of each patient in the IG. During the follow-up period, the patients received short, tailored, individualized motivational feedback directly through the app 1–3 times a week. Additionally, they received comprehensive individual feedback via email once a week for the first 12 weeks and every fourth week for the rest of the year. All feedback was based on what each patient had done, not done or on each patient’s notes. Patients could submit questions to the supervisor at any time and would receive an answer within two working days. Patients were followed for one year by the same supervisor who included the patients at baseline. The same supervisor monitored and gave feedback to all patients in the IG for the whole year. The supervisor was a physiotherapist specialized in cardiovascular and pulmonary physiotherapy with seven years of experience in CR. A more detailed description of the intervention has been previously reported by our group.^15^

The intervention was recently evaluated in a feasibility study and was found to be feasible.^17^ Satisfaction with the technology was high; patients found the intervention both useful and motivational, and time spent on monitoring and giving feedback to each patient was acceptable.^17^

### Outcomes and assessments

The primary outcome was difference in VO_2peak_. Secondary outcome measures included exercise performance, evaluated as time to exhaustion, peak incline (%) and peak velocity (km/h), in addition to body weight, resting BP, blood samples (lipid profile and triglycerides), exercise habits, HRQL, health status and self-perceived goal achievement. All assessments were performed at baseline (post-CR) and after one year at the same CR centre where the patient was recruited. Test personnel measuring the primary outcome were blinded for group allocation. During baseline assessment, performed by the same physiotherapist in all included patients, demographic data were collected. Additionally, all patients chose their own individual goals related to healthy behaviour for the next year and were encouraged to select tasks based on their being able to reach each goal.

### VO_2peak_ and exercise performance

All patients performed a CPET on a treadmill before entering the study, to ensure eligibility to the study and to determine VO_2peak_ and exercise performance. For this purpose, two standardized protocols, a walking and a running protocol, were drafted. During both tests, the patients were strongly encouraged to exercise to exhaustion. Experienced test personnel chose which protocol was most suitable for each patient based on age and physical functioning. The walking protocol started at 3.5 km/h and 0% incline for 2 min, after which the velocity and incline was increased by 0.5 km/h and 1% each minute. If 6 km/h was reached, only the incline increased by 2% each minute. The running protocol started at 5 km/h and 0% inclination for 2 min, whereupon the velocity and incline increased by 1 km/h and 2%, every other minute, respectively. A respiratory exchange ratio of ≥1.1 was used to verify maximal effort. Additionally, the BORG scale (6–20) was used to verify perceived exertion. For comparison, the same test protocol was used both at baseline and at follow-up in all patients. Additionally, we strived to perform the CPETs at the same time of day. Patients were told to take medication and eat and drink as normal before both tests. The highest 30-s VO_2_ measurement was used as VO_2peak_, and exercise performance was evaluated as time to exhaustion, peak incline (%) and peak velocity (km/h). Furthermore, the CPET was performed with continuous 12-lead electrocardiogram monitoring. Pulmonary ventilation and gases (oxygen and carbon dioxide) were recorded breath-by-breath using a Vyntus CPX metabolic analyser (Vyaire Medical, Hôchberg, Germany) at one of the CR centres. At the other CR centre, pulmonary ventilation and gases were analysed using a Schiller Ganshorn ergo-spirometry system (Schiller AB, Baar, Switzerland) at baseline, and with a Vyntus CPX (Customed, Ottobunn, Germany) at follow-up.

### Bodyweight

Bodyweight was measured without shoes, wearing exercise clothes prior to the CPET at both baseline and follow-up. Efforts were made to use the same equipment at both pre- and post-test.

### BP

BP was measured prior to the CPET. Measurement was done manually, preferably on the left arm. Patients relaxed on a chair for 3–5 min before measurements were taken. Three measurements were performed, of which the lowest measured value was used.

### Blood samples

Venous blood was drawn following an overnight fast, using standard local procedures at the patient’s general practitioner, within four weeks prior to CPET. We gathered data on low-density lipoprotein cholesterol, high-density lipoprotein cholesterol, total cholesterol and triglycerides. Patients had to bring the results to baseline and follow-up assessment.

### Exercise habits

Patients were asked for their exercise habits at both assessments. Exercise habits were defined as mean exercise sessions each week for the last year. In this context, an exercise session was defined as structured activity lasting at least 30 min, where you got both sweaty and breathless, and felt like taking a shower afterwards.

### HRQL

HRQL was measured with HeartQoL. This is a disease-specific HRQL questionnaire, found to be both valid and reliable in patients referred to CR.^18–22^ The questionnaire consists of 14 questions, which gives two subscales: physical (10-item) and emotional (4-item) HRQL.^23^ Combining these subscales provides a global scale score.^23^ The score ranges from 0 to 3, where a higher score indicates better HRQL.

### Health status

Health status was measured by EQ-5D. The questionnaire consists of five questions, which represent five different dimensions of health status: mobility, self-care, usual activities, pain/discomfort and anxiety/depression.^24^ Each question gives five answer options, where a score of 1 represents best possible score and 5 represents worst possible score.^24^ In addition, EQ-5D consists of an overall health question (EQ visual analogue scale [VAS]), where the answer is given on a Likert-scale (0–100, where 0 represents the worst possible health and 100 the best possible health).^24^

### Self-perceived goal achievement

Self-perceived goal achievement for each individual goal was assessed on a Likert-scale (0–100, where 0 represented being far away from reaching the goal and 100 meant that the goal had been reached).

### Sample size

Sample size was calculated from the primary outcome, VO_2peak_, assuming a difference in relative VO_2_ between groups of 3.5 ml/kg/min to a clinical important difference.^5–8^ The associated standard deviation (SD) was estimated to be 6 ml/kg/min based on the feasibility study.^17^ With a power of 0.8 and significance level of 0.05, the sample size was calculated to be sufficient with 47 patients in each group. To allow for a 20% dropout, we aimed to include 113 patients in total.

### Statistical analyses

IBM SPSS Statistics (version 25) was used for statistical analysis. Continuous, normally distributed baseline data were analysed with an independent t-test to test for differences between groups, and Pearson’s chi-squared test was used to analyse the categorical data. Baseline differences between cases with and without one-year primary outcome data were analysed using the same statistical tests. The assessment of missing data was done based on strategies for dealing with missing data in clinical trials.^25^ The paired samples *t*-test was used to analyse within-group differences from baseline to follow-up. Primary and secondary outcome measures were analysed for differences between groups using a general linear model with values after intervention as the dependent variable, baseline values as covariates and group as factor (analysis of covariance). Analysis was carried out by intention-to-treat and all tests were two-sided. Data are presented as mean ± SD unless stated otherwise. A *p-*value <0.05 was considered significant.

## Results

**Figure 1.**
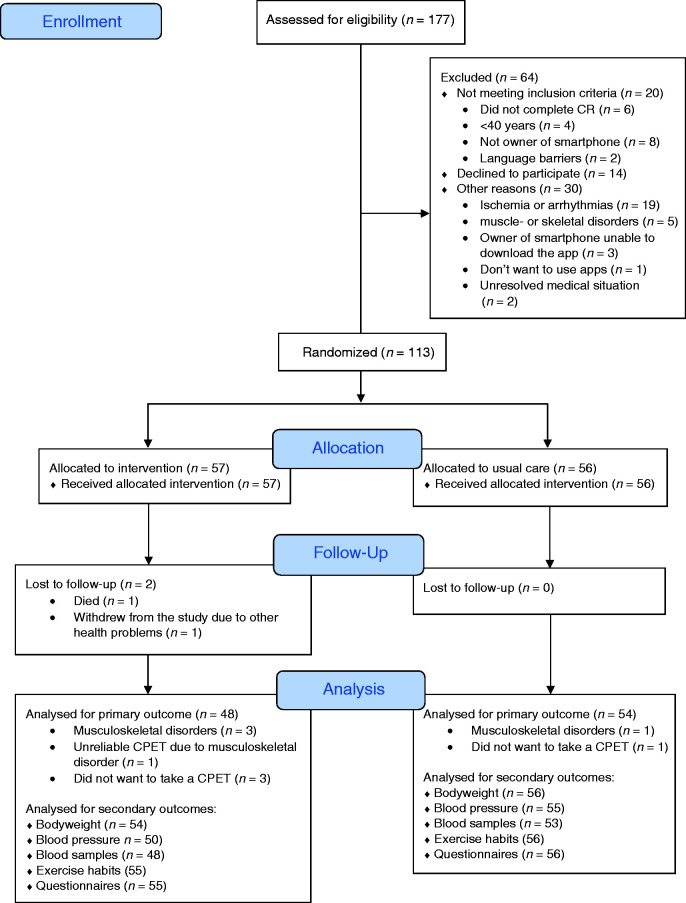
The CONSORT flow diagram. CR: cardiac rehabilitation; CPET: cardiopulmonary exercise test.

Between October 2017 and June 2018, 177 patients at the two CR centres were screened for eligibility. A total of 113 were included and were randomized to the IG or CG (see Figure 1). One-year follow-up was completed in June 2019. The baseline characteristics are presented in Table 1. There were no statistically significant differences in characteristics at baseline between the two groups. There were also no statistically significant differences between the groups in change in medication during follow-up. At baseline, 69 (61.1%) used beta-blockers. During follow-up, 19 (27.5%) patients reduced their beta-blocker dose and four (5.8%) increased the dose. Out of those 55 (48.7%) patients using antihypertensive medication at baseline, six (10.9%) reduced their dose and seven (12.7%) increased it. Statins were used by 96 (85%) patients at baseline; 17 (17.7%) reduced their dose and nine (9.4%) increased their dose. Furthermore, 75 (66.4%) patients used acetylsalicylic acid and plate inhibitors; 39 (52%) patients reduced their dose during follow-up and one (1.3%) patient increased it.

**Table 1.** Patient baseline characteristics.

	Total (*n* = 113)	Control group (*n* = 56)	Intervention group (*n* = 57)
Age	59.0 ± 8.7	58.4 ± 8.2	59.5 ± 9.1
Female, *n* (%)	25 (22.1)	16 (28.6)	9 (15.8)
Non-European, *n* (%)	4 (3.5)	–	4 (7)
Married or cohabitant, *n* (%)	89 (78.8)	43 (76.8)	46 (80.7)
Current smoker, *n* (%)	4 (3.5)	1 (1.8)	3 (5.3)
Bodyweight	90.2 ± 16.9	88.5 ± 17.0	91.8 ± 16.8
Body mass index	29.0 ± 4.9	28.4 ± 4.6	29.6 ± 5.2
Disease, *n* (%)			
Acute coronary syndrome	44 (38.9)	22 (39.3)	22 (38.6)
Coronary artery disease	39 (34.5)	19 (33.9)	20 (35.1)
Valve	19 (16.8)	8 (14.3)	11 (19.3)
Cardiac arrest	1 (0.9)	1 (1.8)	–
Atrial fibrillation	3 (2.7)	1 (1.8)	2 (3.5)
Other	7 (6.2)	5 (8.9)	2 (3.5)
Treatment, *n* (%)			
Percutaneous coronary intervention	55 (48.7)	26 (46.4)	29 (50.9)
Coronary artery bypass graft	22 (19.5)	12 (21.4)	10 (17.5)
Valve surgery	19 (16.8)	8 (14.3)	11 (19.3)
Implantable cardioverter-defibrillator	2 (1.8)	1 (1.8)	1 (1.8)
Pacemaker	2 (1.8)	2 (3.6)	–
Conservatively	10 (8.8)	6 (10.7)	4 (7.0)
Other	3 (2.7)	1 (1.8)	2 (3.5)
Medication, *n* (%)			
Beta-blocker	69 (61.1)	32 (57.1)	37 (64.9)
Statins	96 (85)	45 (80.4)	51 (89.5)
ASA + plate inhibitor	75 (66.4)	39 (69.6)	36 (63.2)
Antihypertensive	55 (48.7)	29 (51.8)	26 (45.6)
Type D personality, *n* (%)	13 (11.5)	8 (14.3)	5 (8.8)
Smartphone, *n* (%)			
iPhone	60 (53.1)	30 (53.6)	30 (52.6)
Android	53 (46.9)	26 (46.4)	27 (47.4)
Type of cardiac rehabilitation, *n* (%)			
One week	35 (31)	17 (30.4)	18 (31.6)
Four weeks	40 (35.4)	20 (35.7)	20 (35.1)
Twelve weeks	38 (33.6)	19 (33.9)	19 (33.3)
Blood samples			
LDL-cholesterol	2.2 ± 0.9	2.3 ± 0.9	2.2 ± 0.8
HDL-cholesterol	1.3 ± 0.4	1.3 ± 0.4	1.2 ± 0.4
Total cholesterol	4.0 ± 1.0	4.0 ± 1.0	4.0 ± 0.9
Triglycerides	1.4 ± 0.9	1.3 ± 1.0	1.5 ± 0.9
Blood pressure			
Systolic blood pressure	135 ± 17	136 ± 18	133 ± 17
Diastolic blood pressure	81 ± 9	81 ± 8	81 ± 10
Exercise habits last year	1.4 ± 1.5	1.3 ± 1.5	1.6 ± 1.5
Cardiopulmonary exercise test			
VO_2peak_ (ml/kg/min)	29.6 ± 7.7	29.9 ± 6.7	29.4 ± 8.7
VO_2peak_ (l/min)	2.64 ± 0.74	2.63 ± 0.75	2.65 ± 0.74
Time to exhaustion (sec)	601 ± 124	603 ± 102	598 ± 144

Values are mean ± standard deviation or *n* (%), unless otherwise stated. Group differences were not significant.

VO_2peak_: peak oxygen uptake; ASA: acetylsalicylic acid; Type D personality: Type D Scale-14, a standard measure of type D personality; LDL-cholesterol: low-density lipoprotein cholesterol; HDL-cholesterol: high-density lipoprotein cholesterol; Exercise habits last year: mean exercise sessions each week for the last year.

Adherence to the app in the IG, defined as use of the app by answered tasks throughout the study period, was high. A total of 71% (*n* = 39) of patients answered all tasks throughout the year, 84% (*n* = 46) answered more than 80% of the tasks and 91% (*n* = 50) answered more than 50% of the tasks.

There were some missing data at follow-up in the primary outcome, especially in the IG (Figure 1). There were no statistically significant differences in the baseline characteristics between cases with missing data in the primary outcome at one-year follow-up and cases with no missing data in the primary outcome. Missing data occurred completely at random, since they were unrelated to any observed or unobserved variables.^25^ Therefore, imputation of missing data was not conducted.^25^

No severe adverse events, defined as acute myocardial infarction or cardiac arrest, were registered during CPETs.

There was a statistically significant difference in both relative and absolute VO_2peak_ between IG and CG from baseline to one-year follow-up, with a mean difference of 2.2 ml/kg/min, 95% confidence interval (CI) 0.9–3.5 (*p* = 0.001) and 0.17 l/min, 95% CI 0.06–0.28 (*p* = 0.002), respectively (Table 2).

**Table 2.** Primary and secondary outcome measures at baseline and at one-year follow-up.

	Control group (*n* = 54)			Intervention group (*n* = 48)		
	Baseline	1 year	Change	Baseline	1 year	Change
Cardiopulmonary exercise test						
VO_2peak_ (ml/kg/min)	29.5 ± 6.5	28.7 ± 6.9	–0.8 ± 3.3	29.8 ± 9.1	31.2 ± 8.8	1.4 ± 3.5^††,^*
VO_2peak_ (l/min)	2.57 ± 0.69	2.48 ± 0.68	–0.09 ± 0.29*	2.63 ± 0.76	2.71 ± 0.74	0.08 ± 0.26^†,^*
Time to exhaustion (sec)	601 ± 102	632 ± 119	31 ± 85*	602 ± 155	675 ± 131	72 ± 92^†,^**
Incline (%)	11.0 ± 3.0	11.8 ± 3.8	0.8 ± 2.6*	10.9 ± 4.4	13.1 ± 4.1	2.2 ± 2.9^†,^**
Speed (km/h)	6.7 ± 1.5	6.8 ± 1.5	0.1 ± 0.2	6.8 ± 1.8	7.0 ± 1.8	0.2 ± 0.4*
Support criteria:						
HR_max_ (beats/min)	161 ± 20	164 ± 20	3 ± 12	160 ± 18	164 ± 17	4 ± 14*
RER_peak_	1.16 ± 0.09	1.20 ± 0.08	0.05 ± 0.10**	1.14 ± 0.11	1.21 ± 0.08	0.07 ± 0.10**
RPE BORG 6–20	17.8 ± 1.0	17.5 ± 1.2	–0.3 ± 1.1	17.7 ± 1.1	17.9 ± 1.1	0.2 ± 1.6
Bodyweight (kg)	88.5 ± 17.0	88.6 ± 17.6	0.1 ± 4.4	92.0 ± 16.9	90.4 ± 16.6	–1.6 ± 4.1*
Resting blood pressure (mmHg)						
Systolic	136 ± 18	145 ± 20	9 ± 20*	134 ± 15	143 ± 19	9 ± 17**
Diastolic	81 ± 8	84 ± 11	3 ± 10*	81 ± 9	86 ± 10	5 ± 11*
Blood samples						
LDL-cholesterol	2.2 ± 0.9	2.3 ± 0.9	0.1 ± 0.5	2.2 ± 0.9	2.2 ± 1.0	0 ± 0.8
HDL-cholesterol	1.3 ± 0.4	1.4 ± 0.5	0.1 ± 0.3*	1.2 ± 0.4	1.3 ± 0.4	0 ± .02*
Total cholesterol	4.0 ± 1.0	3.9 ± 1.0	–0.1 ± 0.6	4.1 ± 0.9	4.1 ± 1.0	0 ± 0.9
Triglycerides	1.3 ± 1.0	1.2 ± 0.7	–0.1 ± 0.9	1.6 ± 0.9	1.6 ± 1.6	0 ± 1.1
Exercise habits	1.3 ± 1.5	1.9 ± 1.6	0.6 ± 1.1**	1.6 ± 1.6	3.0 ± 1.9	1.4 ± 1.5^††,^**
HeartQol	2.48 ± 0.54	2.57 ± 0.51	0.09 ± 0.45	2.43 ± 0.59	2.64 ± 0.51	0.21 ± 0.47*
Physical	2.30 ± 0.68	2.31 ± 0.68	0.01 ± 0.57	2.43 ± 0.58	2.52 ± 0.65	0.09 ± 0.54
Emotional	2.44 ± 0.55	2.49 ± 0.49	0.05 ± 0.45	2.43 ± 0.54	2.61 ± 0.51	0.18 ± 0.43*
Global						
EQ VAS	72 ± 14	75 ± 12	3 ± 16	69 ± 18	78 ± 16	9 ± 16**
Self-perceived goal achievement						
Goal 1	62 ± 29	66 ± 32	3 ± 32	54 ± 35	72 ± 29	18 ± 25^†,^**
Goal 2	56 ± 29	50 ± 36	–5 ± 41	43 ± 29	69 ± 31	26 ± 37^†,^**

*n* is given for primary outcome.

Change from baseline to one-year follow-up between group: †*p* < 0.05, ††*p* < 0.001; within group: *p < 0.05, **p < 0.001.

VO_2peak_: peak oxygen uptake; HR_max_: maximal heart rate; RER_peak_: peak respiratory exchange ratio; RPE: rate of perceived exertion; LDL-cholesterol: low-density lipoprotein cholesterol; HDL-cholesterol: high-density lipoprotein cholesterol.

Statistically significant differences between the groups emerged in three of the secondary outcomes: exercise performance, exercise habits and self-perceived goal achievement. Time to exhaustion and peak incline were statistically significant, with a mean difference between groups of 41 s, 95% CI 9–73 (*p* = 0.013) and 1.3%, 95% CI 0.3–2.4 (*p* = 0.014), respectively. Exercise habits increased in both groups from baseline to one-year follow-up, and there was a statistically significant difference between the two groups in favour of the IG, with a mean difference of 0.9 exercise sessions each week, 95% CI 0.4–1.4 (*p* < 0.001). Mean difference in self-perceived goal achievement was 10 points, 95% CI 1–20 (*p* = 0.034) for Goal 1, and 22 points, 95% CI 4–40 (*p* = 0.016) for Goal 2. In total, there were only seven patients that had three goals. Statistical analysis for the third goal has, therefore, not been conducted. There were no significant differences between the two groups from baseline to follow-up in any of the other secondary outcomes. The distribution of scores in EQ-5D at baseline and one-year follow-up are presented in supplementary Table S1. All outcome measures are presented in Table 2.

## Discussion

To our knowledge, the present study is the first to evaluate the effects of individualized follow-up with an app for one year post-CR. Our main finding was a statistically significant difference in favour of the IG in VO_2peak_ as well as exercise performance, exercise habits and self-perceived goal achievement. A strength of our study was that the follow-up time was long, and in line with the time it has been shown to establish or automate a habit.^26^ Another strength of the study is that exercise capacity was measured objectively in all patients. Additionally, despite the long follow-up time, there were few dropouts (*n* = 2).

In the present study, we found a significant mean difference in relative VO_2peak_ of 2.2 ml/kg/min between the groups, from baseline to follow-up. This difference in relative VO_2peak_ between the groups, which was mainly due to the significant difference in absolute VO_2peak_ (0.17 l/min), is supported by a significant difference in exercise performance. It is worth discussing whether the observed difference in VO_2peak_ is clinically relevant. Power calculation was conducted based on 3.5 ml/kg/min as a clinically relevant difference between the two groups.^5–8^ Based on a previous study of a similar Norwegian population,^27^ this was a realistic achievement. In the study by Aamot et al.,^27^ patients were tested one year post-CR without any intervention. They found a mean decline in VO_2peak_ of 1.8 ml/kg/min. As a part of this, we anticipated a greater decline in VO_2peak_ in the CG. We are aware that a difference of 3.5 ml/kg/min may be an optimistic difference in a follow-up study as patients, on average, improve their VO_2peak_ by ≥3.5 ml/kg/min during participation in 12-week high-intensity, interval-based CR programmes.^27,28^ In a study by Keteyian et al., a difference of 1 ml/kg/min was shown to be clinically relevant, with a 15% reduction in CVD- and all-cause mortality.^29^ The difference of 2.2 ml/kg/min in the present study may, therefore, be important.

In the present study, there were significant differences between the groups in some of the secondary outcome measures. Patients in the IG reported that they exercised significantly more post-CR compared to patients in the CG. This is not surprising as self-monitoring, specific goal setting, identifying barriers and developing plans as well as feedback were integrated in the intervention, and these behavioural change techniques are frequently associated with positive physical activity outcomes in a post-CR setting.^30^ The statistically significant difference between groups in self-perceived goal achievement can be explained by the fact that most patients had exercise-related goals, as well as by the fact that patients in the IG increased both VO_2peak_ and exercise performance significantly more than those in the CG. Contrary to expectations, we did not find a statistically significant difference in bodyweight between the two groups at follow-up. We still think it is worth pointing out the mean reduction of 1.6 kg in the IG, as every kilogram of weight loss in a lifestyle intervention has shown a 16% reduction of diabetes type 2 incidence.^31^ In BP, lipid profile, triglycerides, HRQL and health status, there were no significant differences between the groups. These findings correspond with Madssen et al., who found that HRQL, BP, lipids and triglycerides were maintained one-year post-CR, independent of whether they were followed by a maintenance exercise programme or received usual care post-CR.^32^ These corresponding findings may indicate a need for optimization of interventions post-CR in order to influence important outcome measures such as HRQL, BP and lipids.

The effectiveness of mhealth interventions, such as apps, to monitor and motivate individuals to adhere to lifestyle behaviours established or initiated in CR has been sparingly evaluated and there appears to be a knowledge gap.^1^ Apps have been evaluated in the CR population in the form of a home-based CR model to increase CR uptake, adherence and completion,^33^ and as an adjunct to CR to improve risk factor profiles and lifestyle behaviours.^34^ In these settings, the use of apps has shown promising results. Recently, a systematic review and meta-analyses evaluating the impact of wearable physical activity monitoring devices in the maintenance phase of CR were published.^14^ Three of the nine included studies in this systematic review measured VO_2peak_,^35–37^ and the overall mean difference was 2.24 ml/kg/min, 95% CI 0.58–3.89,^14^ which is similar to our result. However, the total number of participants included in this meta-analysis (*n* = 133) was just above the number of participants included in our analyses (*n* = 102), and time to follow-up ranged from 12 weeks^35,36^ to six months.^37^

The use of technology in follow-up of patients post-CR seems to be promising. However, it appears that it is still in its early days. It is, therefore, crucial to learn from previous research to be able to develop and establish the ultimate post-CR mhealth programme. As pointed out in an editorial (by Hugo Saner) some years ago,^38^ regardless of the type of technology used, those involved in new technologies must keep in mind that the patients’ individual needs are of primary importance. The use of reminders, such as those incorporated in the app used in the present study, has shown to be beneficial in mhealth interventions for secondary prevention in CVD.^10^ Additionally, specific goal setting, developing plans and feedback are known techniques associated with positive physical activity outcomes in a post-CR setting.^30^ In this context, we find it both relevant and important to point out the assumed importance of having a real person monitoring and giving feedback to the patients. Patients in the present study reported (unpublished data) that they wanted to respond precisely to the app because they knew that the supervisor monitored all the answers and notes. They felt they answered directly to the supervisor rather than answering to an app database or robot. This may have been important for the demonstrated effects as the relationship between health staff and patient has shown to influence clinical outcomes in patients with type 2 diabetes^39^ and adherence to lifestyle change in patients with hypertension.^40^ This might also explain the high levels of use of the app, where 84% (*n* = 46) answered more than 80% of the tasks throughout the year. The high level of use is impressive, as technological problems often appear as a reason for drop-outs, such as in the comparable study by Skobel et al.,^37^ where only 30% of the IG remained at follow-up. At this point, we can only speculate on the reasons for the high use of the app in our study. However, we believe that the high level of individualization, having a real person behind the app as well as quite simple technology may have been crucial.

## Limitations

One limitation in this study is the difference in CPET equipment used at baseline and follow-up for all patients recruited from one of the CR centres. However, as all patients were tested with the same CPET equipment at baseline and follow-up, measurement differences that may have occurred due to different test equipment will be the same for both groups. In this sense, it is a strength that we also measured exercise performance. Additionally, although the inclusion criteria for this study were quite broad and made most patients at CR eligible for participation in the study, mainly patients with CAD were included. This must be considered when interpreting the results. Different subgroups of patients completing CR could benefit differently from the intervention, and this could not be tested in this limited sample. Future research could narrow the inclusion criteria or increase the sample size substantially to allow for systematic subgroup analyses.

## Conclusion

Individualized follow-up enabled with an app for one year is effective to improve VO_2peak_, exercise performance, exercise habits and self-perceived goal achievement in patients post-CR. Since the automation of new habits is a process over time, any long-term effects of such follow-up should be evaluated 2–5 years after the intervention has ended. Then we will be further able to fill the knowledge gap related to long-term adherence to an active and healthy lifestyle post-CR.

## Supplemental Material

CPR905717 Supplemental Material1 - Supplemental material for Long-term follow-up with a smartphone application improves exercise capacity post cardiac rehabilitation: A randomized controlled trial Click here for additional data file. Supplemental material, CPR905717 Supplemental Material1 for Long-term follow-up with a smartphone application improves exercise capacity post cardiac rehabilitation: A randomized controlled trial by Pernille Lunde, Asta Bye, Astrid Bergland, Jostein Grimsmo, Even Jarstad and Birgitta Blakstad Nilsson in European Journal of Preventive Cardiology

CPR905717 Supplemental Material2 - Supplemental material for Long-term follow-up with a smartphone application improves exercise capacity post cardiac rehabilitation: A randomized controlled trial Click here for additional data file. Supplemental material, CPR905717 Supplemental Material2 for Long-term follow-up with a smartphone application improves exercise capacity post cardiac rehabilitation: A randomized controlled trial by Pernille Lunde, Asta Bye, Astrid Bergland, Jostein Grimsmo, Even Jarstad and Birgitta Blakstad Nilsson in European Journal of Preventive Cardiology

CPR905717 Supplemental Material3 - Supplemental material for Long-term follow-up with a smartphone application improves exercise capacity post cardiac rehabilitation: A randomized controlled trial Click here for additional data file. Supplemental material, CPR905717 Supplemental Material3 for Long-term follow-up with a smartphone application improves exercise capacity post cardiac rehabilitation: A randomized controlled trial by Pernille Lunde, Asta Bye, Astrid Bergland, Jostein Grimsmo, Even Jarstad and Birgitta Blakstad Nilsson in European Journal of Preventive Cardiology
