# Supplementary material for: Long-term follow-up with a smartphone application improves exercise capacity post cardiac rehabilitation: A randomized controlled trial
Source: Eur J Prev Cardiol. 2020 Feb 28;27(16):1782–92. doi: 10.1177/2047487320905717 (PMC7564298; doi:10.1177/2047487320905717)
Supplement: CPR905717 Supplemental Material1 - Supplemental material for Long-term follow-up with a smartphone application improves exercise capacity post cardiac rehabilitation: A randomized controlled trial [file CPR905717_Supplemental_Material1.pdf]

**Tabel S1.** Distribution in percentage of reporting level 1 to 5 by dimension, group and measure time point							
**EQ-5D DIMENSION**		Total		Control group		Intervention group	
		Baseline (n=113)	1-year (n=111)	Baseline (n=56)	1-year (n=56)	Baseline (n=57)	1-year (n=55)
MOBILITY	Level 1	85.8%	92.8%	85.7%	89.3%	86%	96.4%
	Level 2	10.6%	4.5%	8.9%	5.4%	12.3%	3.6%
	Level 3	2.7%	1.8%	5.4%	3.6%	0%	0%
	Level 4	0.9%	0.9%	0%	1.8%	1.8%	0%
	Level 5	0%	0%	0%	0%	0%	0%
SELF-CARE	Level 1	95.6%	98.2%	92.9%	98.2%	98.2%	98.2%
	Level 2	4.4%	1.8%	7.1%	1.8%	1.8%	1.8%
	Level 3	0%	0%	0%	0%	0%	0%
	Level 4	0%	0%	0%	0%	0%	0%
	Level 5	0%	0%	0%	0%	0%	0%
USUAL ACTIVITY	Level 1	74.3%	82.9%	80.4%	85.7%	68.4%	80%
	Level 2	21.2%	16.2%	16.1%	12.5%	26.3%	20%
	Level 3	4.4%	0.9%	3.6%	1.8%	5.3%	0%
	Level 4	0%	0%	0%	0%	0%	0%
	Level 5	0%	0%	0%	0%	0%	0%
PAIN / DISCOMFORT	Level 1	40.7%	50.5%	42.9%	46.4%	38.6%	54.5%
	Level 2	47.8%	31.5%	44.6%	32.1%	50.9%	30.9%
	Level 3	8%	12.6%	7.1%	12.5%	8.8%	12.7%
	Level 4	2.7%	5.4%	3.6%	8.9%	1.8%	1.8%
	Level 5	0.9%	0%	1.8%	0%	0%	0%
ANXIETY / DEPRESSION	Level 1	60.2%	64.9%	55.4%	60.7%	64.9%	69.1%
	Level 2	36.3%	28.8%	42.9%	33.9%	29.8%	23.6%
	Level 3	3.5%	4.5%	1.8%	1.8%	5.3%	7.3%
	Level 4	0%	1.8%	0%	3.6%	0%	0%
	Level 5	0%	0%	0%	0%	0%	0%
