# Supplementary material for: Long-term follow-up with a smartphone application improves exercise capacity post cardiac rehabilitation: A randomized controlled trial
Source: Eur J Prev Cardiol. 2020 Feb 28;27(16):1782–92. doi: 10.1177/2047487320905717 (PMC7564298; doi:10.1177/2047487320905717)
Supplement: CPR905717 Supplemental Material2 - Supplemental material for Long-term follow-up with a smartphone application improves exercise capacity post cardiac rehabilitation: A randomized controlled trial [file CPR905717_Supplemental_Material2.pdf]

**Figure 1.** The CONSORT flow diagram

CR: cardiac rehabilitation, CPET: cardiopulmonary exercise test
