# Supplementary figures and images for: Long-term follow-up with a smartphone application improves exercise capacity post cardiac rehabilitation: A randomized controlled trial
Source: Eur J Prev Cardiol. 2020 Feb 28;27(16):1782–92. doi: 10.1177/2047487320905717 (PMC7564298; doi:10.1177/2047487320905717)

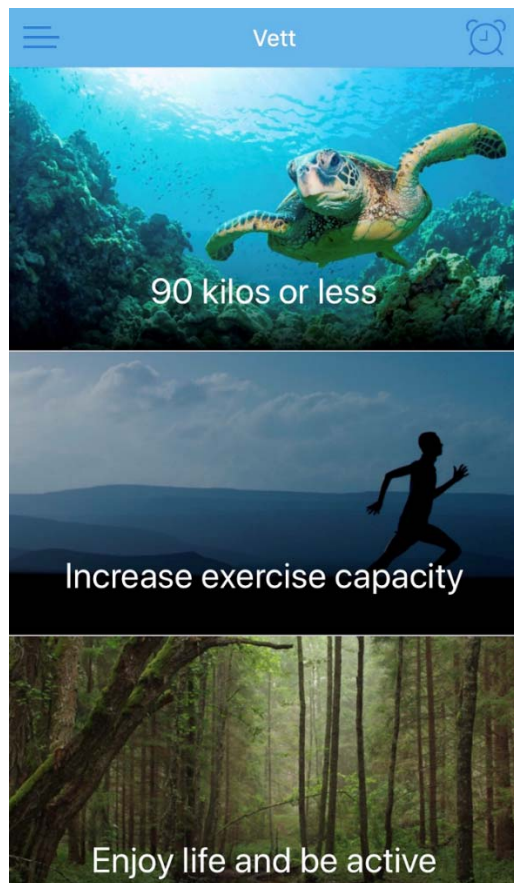

Figure S1. Example of user interface of the app showing individual goals

Supplement: CPR905717 Supplemental Material3 - Supplemental material for Long-term follow-up with a smartphone application improves exercise capacity post cardiac rehabilitation: A randomized controlled trial [file CPR905717_Supplemental_Material3.pdf]
